# High-level expression and characterization of a novel cutinase from *Malbranchea cinnamomea* suitable for butyl butyrate production

**DOI:** 10.1186/s13068-017-0912-z

**Published:** 2017-09-19

**Authors:** Xiaojie Duan, Yu Liu, Xin You, Zhengqiang Jiang, Shaoxiang Yang, Shaoqing Yang

**Affiliations:** 1 Beijing Advanced Innovation Center for Food Nutrition and Human Health, China Agricultural University, Beijing, 100083 China; 2 College of Food Science and Nutritional Engineering, China Agricultural University, Beijing, 100083 China; 3 College of Engineering, China Agricultural University, Beijing, 100083 China; 4 Beijing Key Laboratory of Flavor Chemistry, Beijing Technology and Business University (BTBU), Beijing, 100048 China

**Keywords:** Cutinase, *Malbranchea cinnamomea*, High-level expression, Stability, Butyl butyrate, Crystal structure

## Abstract

**Background:**

Butyl butyrate has been considered as a promising fuel source because it is a kind of natural ester which can be converted from renewable and sustainable lignocellulosic biomass. Compared with the conventional chemical methods for butyl butyrate production, the enzymatic approach has been demonstrated to be more attractive, mainly owing to the mild reaction conditions, high specificity, low energy consumption, and environmental friendliness. Cutinases play an important role in the butyl butyrate production process. However, the production level of cutinases is still relatively low. Thus, to identify novel cutinases suitable for butyl butyrate synthesis and enhance their yields is of great value in biofuel industry.

**Results:**

A novel cutinase gene (*McCut*) was cloned from a thermophilic fungus *Malbranchea cinnamomea* and expressed in *Pichia pastoris*. The highest cutinase activity of 12, 536 U/mL was achieved in 5-L fermentor, which is by far the highest production for a cutinase. McCut was optimally active at pH 8.0 and 45 °C. It exhibited excellent stability within the pH range of 3.0–10.5 and up to 75 °C. The cutinase displayed broad substrate specificity with the highest activity towards *p*-nitrophenyl butyrate and tributyrin. It was capable of hydrolyzing cutin, polycaprolactone, and poly(butylene succinate). Moreover, McCut efficiently synthesized butyl butyrate with a maximum esterification efficiency of 96.9% at 4 h. The overall structure of McCut was resolved as a typical α/β-hydrolase fold. The structural differences between McCut and *Aspergillus oryzae* cutinase in groove and loop provide valuable information for redesign of McCut. These excellent features make it useful in biosynthesis and biodegradation fields.

**Conclusions:**

A novel cutinase from *M. cinnamomea* was identified and characterized for the first time. High-level expression by *P. pastoris* is by far the highest for a cutinase. The enzyme exhibited excellent stability and high esterification efficiency for butyl butyrate production, which may make it a good candidate in biofuel and chemical industries.

## Background

With the increasing gradual energy consumption, depletion of fossil fuel, and growing environmental awareness, development of alternative renewable energy sources has received considerable attention [1–3]. Short-chain butyrate esters have been considered as potential replacements for aviation kerosene, mineral diesel, or petrol recently because they are natural esters which can be converted from renewable and sustainable lignocellulosic biomass [2]. Among them, butyl butyrate has been accepted as a better alternative as it was found to have some special properties, such as high boiling point, low viscosity, low-temperature behavior, and good miscibility with other fuels [2, 4, 5].

The conventional chemical method for butyl butyrate production has some disadvantages, including high energy consumption, hazardous conditions, and release of a large amount of environmental pollutants [5, 6]. Recently, an enzymatic approach for butyl butyrate production has been developed and further demonstrated to be more promising than those chemical routes, mainly owing to the mild reaction conditions, high specificity, low energy consumption, and environmental friendliness [5]. In the bioconversion process, lipases have been used as the major enzymes for the synthesis of butyl butyrate [4–7]. However, cutinases may be more suitable and effective in butyl butyrate synthesis since they typically show preference for short-chain length substrates, while lipases usually have higher affinity for long-chain substrates [8]. Hence, the identification of novel cutinases suitable for butyl butyrate production is of great value.

Cutinases (EC 3.1.1.74) are the smallest member of α/β hydrolase family, capable of catalyzing not only hydrolysis reactions, but also esterification and transesterification reactions [9]. So far, a number of cutinases have been investigated from fungi and bacteria [9]. In general, enzymes from thermophilic microorganisms display relatively high optimal temperature and good thermostability compared to those from mesophilic microorganisms. However, there are few reports on cutinases from thermophilic fungi, except for *Humicola insolens* [10] and *Thielavia terrestris* [11, 12]. Although many cutinase genes have been cloned and expressed, few reports are available on bioreactor systems for large-scale production [8, 13–16]. Additionally, the production level of cutinases is still relatively low. The *Thermobifida fusca* cutinase expressed in *Escherichia coli* represented the highest yield ever reported, with an activity of 2258.5 U/mL and a protein concentration of 5.1 g/L [16]. To meet the needs of industrial applications and reduce production cost, high-level expression of cutinases is urgent and necessary. *Pichia pastoris* is an excellent protein expression host, which has been widely used for the production of various proteins, including industrial enzymes [17], and human interferon gamma [18, 19]. However, no cutinase gene has been ever highly expressed in *P. pastoris* suitable for commercial production.

Up to now, three-dimensional structures of several cutinases have been deposited in the Protein Data Bank, including six fungal and two bacterial cutinases. The crystal structure of the cutinase from *Fusarium solani pisi* was firstly determined and thoroughly studied [20, 21]. Structures of fungal cutinases from *Aspergillus oryzae* [22], *Fusarium oxysporum* [23], *Glomerella cingulata* [24], *H. insolens* [10], and *Trichoderma reesei* [25], and two bacterial cutinases from *Thermobifida alba* [26] and *T. fusca* [27] have been solved. Structural studies of cutinases can help further understand their action modes and obtain more useful information for the redesign of enzymes to fulfill industrial application requirements.

The thermophilic fungus, *Malbranchea cinnamomea* S168, has been found to produce glycoside hydrolases such as α-amylase and xylanase [28, 29]. The secretome analysis showed that *M. cinnamomea* produced cutinase [30]. However, no cutinase from *M. cinnamomea* has been ever studied. In this paper, a novel cutinase gene (*McCut*) from *M. cinnamomea* S168 was cloned and efficiently expressed in *P. pastoris*. The recombinant cutinase was purified, biochemically and structurally characterized, and its application in butyl butyrate synthesis was further investigated.

## Results

### Cloning and sequence analysis of a cutinase gene from *M. cinnamomea*

A cutinase gene (*McCut*) was amplified from cDNA, and it contains an open reading frame of 648 bp, encoding 215 amino acids. There are two introns of 61 and 108 bp, respectively, in the cutinase gene. The mature protein has a predicted molecular mass of 21,180 Da and a theoretical *p*I of 5.19. The N-terminal region contained a predicted signal peptide of 16 amino acids, and the protein sequence did not have *N*-glycosylation site. The gene sequence has been submitted to GenBank under the Accession Number KY568910.1.

**Fig. 1:**
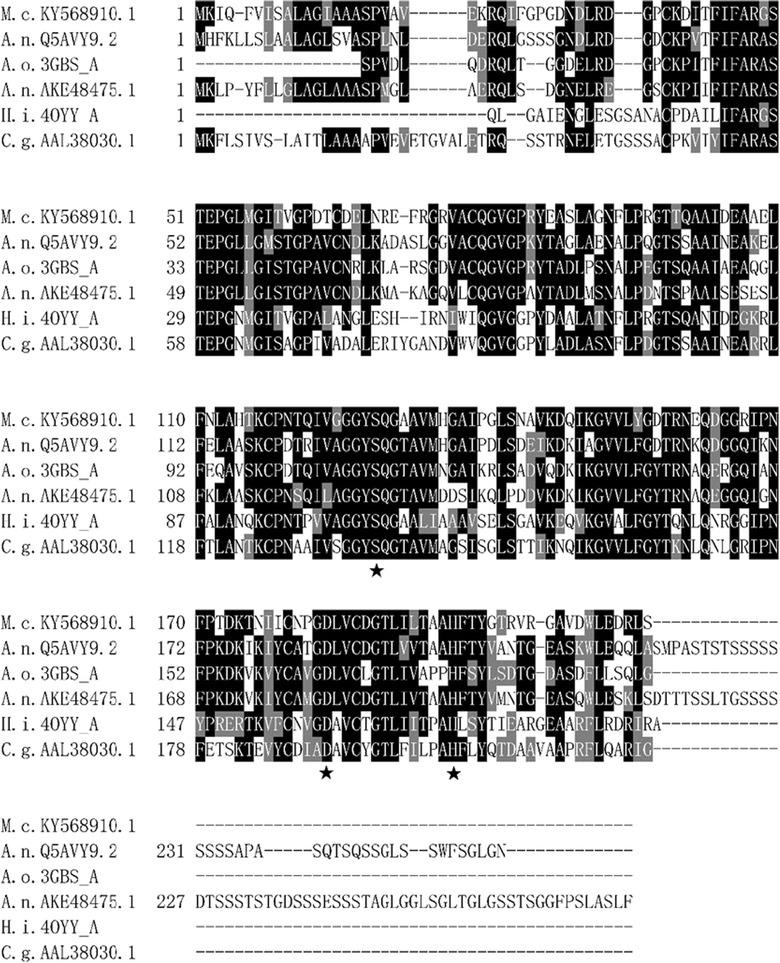
Multiple alignment of amino acid sequences of McCut with homologous cutinases. Numbers on the left are the residue number of the first amino acid in each line. The putative catalytic triad (denoted by stars below the residues) of McCut was predicted by alignment with structure-resolved *A. oryzae* cutinase (PDB ID: 3GBS_A). Abbreviations and GenBank Accession Numbers or PDB ID of the cutinases in the alignment are as follows: *M. cinnamomea* S168 (M.c.KY568910.1), *A. nidulans* FGSC A4 (A.n.Q5AVY9.2), *A. oryzae* (A.o.3GBS_A), *A. niger* (A.n.AKE48475.1), *H. insolens* (H.i.4OYY_A), and *Colletotrichum gloeosporioides* (C.g.AAL38030.1). Identical residues are shaded in black, and conserved residues are shaded in gray

Sequence analysis revealed that McCut contained a catalytic triad consisting of Ser128, Asp183, and His196. According to the sequence similarity analysis, the deduced amino acid sequence of McCut shared the highest identity of 65% with the cutinase from *Aspergillus nidulans* (Q5AVY9.2), followed by cutinases from *A. oryzae* (63%, 3GBS_A), *Aspergillus niger* (59%, AKE48475.1), *H. insolens* (54%, 4OYY_A), and *Colletotrichum gloeosporioides* (53%, AAL38030.1) (Fig. 1).

### Expression of the cutinase gene in *P. pastoris* by high-cell density fermentation

**Fig. 2:**
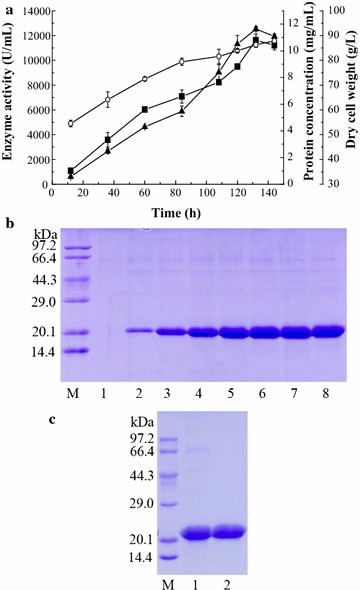
Time course of recombinant cutinase produced by *P. pastoris* in a 5-L fermentor (**a**), and SDS-PAGE analysis of expression (**b**) and purification steps (**c**). The enzyme activity (filled triangle) and protein concentration (filled square) were monitored during high-cell density cultivation. The cutinase activity was determined at 45 °C in 50 mM Tris–HCl (pH 8.0) using *p*NPB as the substrate. All data are mean values of triplicate measurements. In **b**, lane M: low-molecular weight standard protein markers; lane 1: before methanol induction; lanes 2–8: culture supernatant collected after 12, 36, 60, 84, 108, 120, 132, and 144 h of methanol induction, respectively. In **c**, lane M: low-molecular weight standards; lane 1: crude enzyme; lane 2: purified enzyme

The cutinase (McCut) was expressed in *P. pastoris* under the *AOX1* promoter. A transformant showing the highest cutinase activity in shake-flask was screened out from YPD plates with a geneticin 418 (G418) concentration of 1 mg/mL and cultivated in a 5-L fermentor. A maximum activity of 12, 536 U/mL was achieved after 132 h, with a protein content of 10.8 g/L and a dry cell weight (dcw) of 86.3 g/L (Fig. 2a). It resulted in the yields of 77, 846 U/g dcw and 14, 500 U/g methanol. Furthermore, the recombinant cutinase was slightly cell wall localized (about 4% of the supernatant activity). From the SDS-PAGE analysis, a very less amount of other proteins was secreted into the medium (Fig. 2b).

### Purification of the recombinant cutinase

McCut was purified to apparent homogeneity by a single step of ion-exchange chromatography using Q Sepharose Fast Flow (QSFF) with a specific activity of 1, 181.6 U/mg and a recovery yield of 88.3%. The purified enzyme showed a single band in SDS-PAGE with a molecular mass of 21.9 kDa (Fig. 2c), which matches with the predicted molecular mass of 21.18 kDa.

### Crystal structure of McCut

**Fig. 3:**
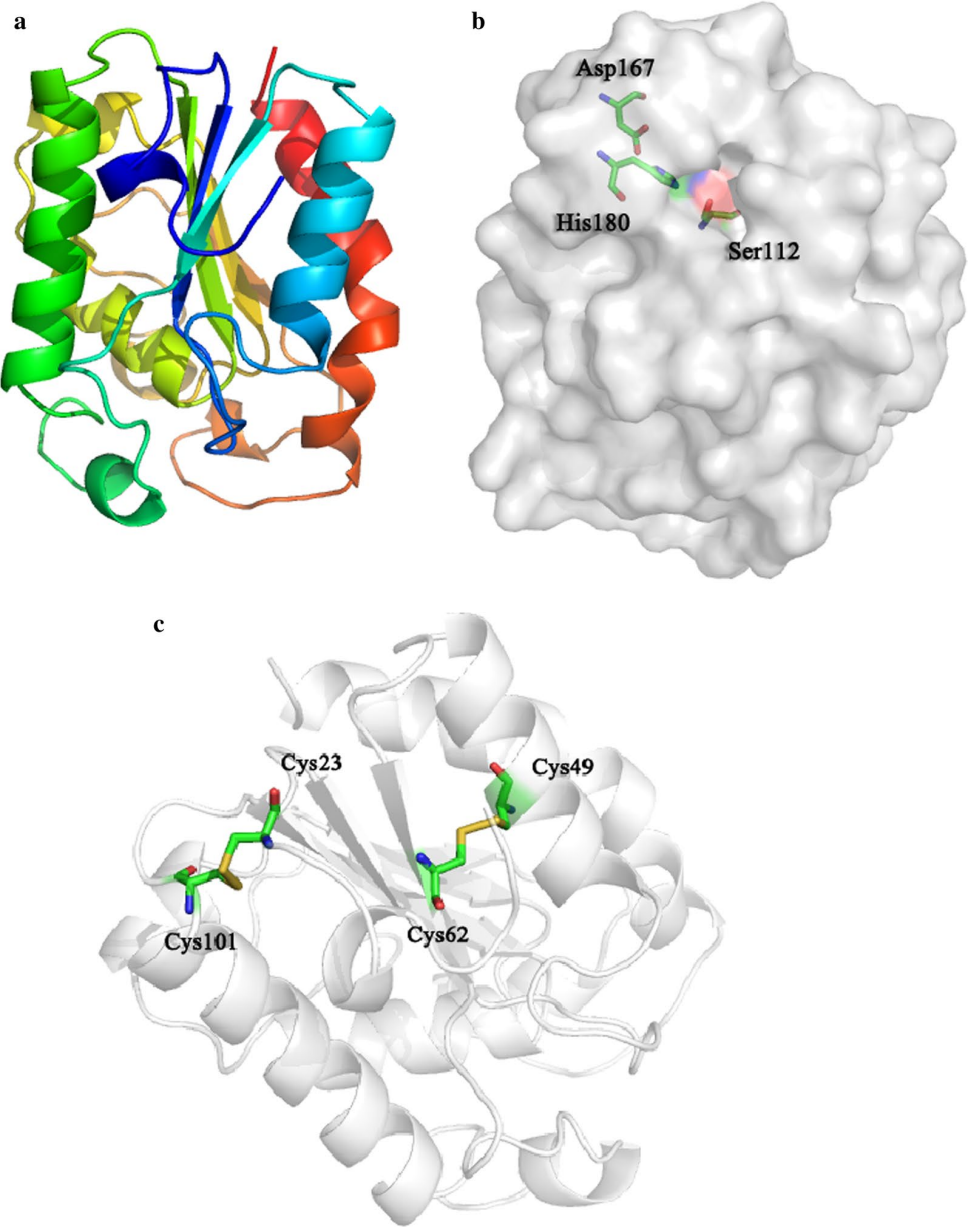
Overall structure of McCut. **a** Overall structure of McCut in the form of a cartoon. It contains five parallel strands and nine α-helices. **b** The molecule with three catalytic sites Ser112, Asp167, and His180 is shown as a surface. **c** Two disulfide bonds between Cys49 and Cys62, and Cys23 and Cys101 are displayed

The crystal structure of McCut was determined at 1.76 Å resolution in space group *P*2_1_22_1_. The *R*_work_ and *R*_free_ were 17.17 and 17.54%, respectively. The crystallographic asymmetric unit contains one protein molecule. McCut is an α/β hydrolase with a central β-sheet of five parallel strands surrounded by nine α-helices (Fig. 3a). The active site of mature protein is composed of the catalytic triad Ser112, Asp167, and His180 (Fig. 3b), which is in accordance with the aforementioned result. There are two disulfide bonds in the cutinase, one between cysteines 49 and 62 and the other linking cysteines 23 and 101 (Fig. 3c).

**Fig. 4:**
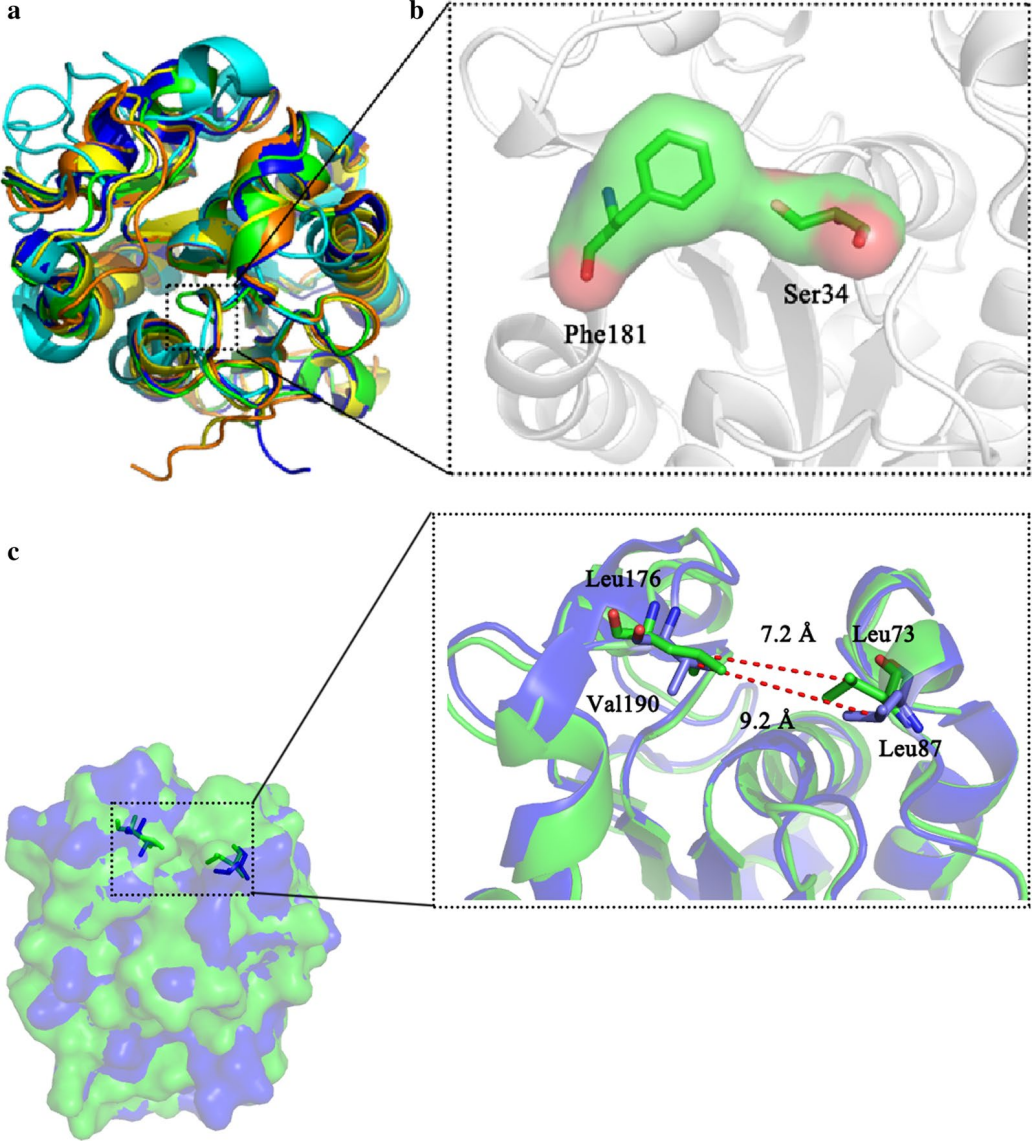
Structural comparison of McCut and other cutinases. **a** Superposition of McCut on other cutinases is shown by ribbon diagram, with ribbons colored according to each enzyme: McCut in green, AoCut (PDB code: 3GBS) in blue, FsCut (PDB code: 1CEX) in yellow, CLE (PDB code: 2CZQ) in cyan, and *G. cingulata* cutinase (PDB code: 3DCN) in orange. **b** The unique loop is further shown as a cartoon in the comparison structure. The difference of Ser34 in McCut leads to the space steric hindrance. **c** The gatekeeper residues Leu73 and Leu176 in McCut, and Leu87 and Val190 in AoCut are shown

Superimposition of the structures of McCut, *A. oryzae* cutinase (AoCut, PDB code: 3GBS), *F. solani* cutinase (FsCut, PDB code: 1CEX), cutinase-like enzyme from *Cryptococcus* sp. S-2 (CLE, PDB code: 2CZQ), and *G. cingulata* cutinase (PDB code: 3DCN) reveals that the overall fold is almost identical (Fig. 4a). The minor difference between McCut and the others can be seen in the loop region (Fig. 4a). The side chains of Ser34 and Phe181 extend to the catalytic groove leading to the space steric hindrance (Fig. 4b). Moreover, two gatekeeper residues, Leu73 and Leu176, in McCut are 7.2 Å apart, whereas the corresponding residues (Leu87 to Val190) of AoCut are separated by 9.2 Å (Fig. 4c). This suggests that McCut has a narrower catalytic cleft.

### Biochemical properties of McCut

**Fig. 5:**
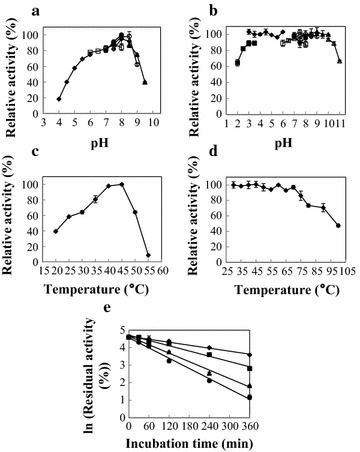
Optimal pH (**a**), pH stability (**b**), optimal temperature (**c**), thermostability (**d**), and thermal denaturation half-lives (**e**) of McCut. The optimal pH was determined at 45 °C in 50 mM of different buffers. To determine pH stability, the enzyme was incubated in various buffers at 50 °C for 30 min, and the residual activity was measured. Buffers used were as follows: glycine–HCl (filled square), pH 2.0–3.5; citrate (filled diamond), pH 3.0–6.0; phosphate (open square), pH 6.0–8.0; Tris–HCl (open circle), pH 7.0–9.0; HEPES (filled circle), pH 7.0–8.0; Tricine (open diamond), pH 7.5–8.5; CHES (filled triangle), pH 8.0–10.0; and CAPS (open triangle), pH 10.0–11.0. For optimal temperature, the enzyme activity was assayed at temperatures ranging from 20 to 55 °C in 50 mM Tris–HCl (pH 8.0). The thermostability was investigated by incubating the enzyme at 30–100 °C in 50 mM Tris–HCl (pH 8.0) for 30 min. The residual activity was measured as described in the “Methods” section. For determination of thermal denaturation half-lives, McCut was incubated at different temperatures in 50 mM Tris–HCl (pH 8.0) for 6 h, and the residual activities at different time points were measured. The temperatures used were 70 °C (filled diamond), 75 °C (filled square), 80 °C (filled triangle), and 85 °C (filled circle). All data are mean values of triplicate measurements

The optimal pH of McCut was found to be pH 8.0 (Fig. 5a). It was stable in a broad pH range from pH 3.0 to 10.5 (Fig. 5b). McCut displayed maximal activity at 45 °C (Fig. 5c). It retained more than 85% of its initial activity after incubation at 75 °C for 30 min (Fig. 5d). Specially, approximately 47.5% of its activity was maintained after incubation at 100 °C for 30 min (Fig. 5d). The half-lives of McCut at 70, 75, 80, and 85 °C were 263, 160, 92, and 67 min, respectively (Fig. 5e).

The influence of different organic solvents on enzyme activity was investigated (Table 1). Short-chain alcohols and acids inhibited the enzyme activity to different degrees. McCut was hardly affected by hexane and isooctane, but enhanced by cyclohexane (127.9%), *n*-heptane (131.3%), and *n*-octane (143.5%). However, the activity was slightly inhibited by acetone, dimethyl sulfoxide, and acetonitrile.

**Table 1:** Effect of organic solvents and surfactants on the enzyme activity of McCut

Solvent	Specific activity (U/mg)	Relative activity (%)
Organic solvent (30%)		
Control	1115.4 ± 11.5	100
Methanol	891.6 ± 4.6^a^	79.9
Ethanol	811.6 ± 43.9^a^	72.8
Isopropanol	908.8 ± 40.4^a^	81.5
Butanol	656.4 ± 16.2^a^	58.8
Acetic acid	649.1 ± 17.3^a^	58.2
Butyric acid	398.3 ± 18.5^a^	35.7
*n*-Hexane	1090.1 ± 35.8	97.7
Cyclohexane	1426.6 ± 32.8^a^	127.9
*n*-Heptane	1465.0 ± 11.6^a^	131.3
Octane	1601.1 ± 8.2^a^	143.5
Isooctane	1089.3 ± 39.3	97.7
Acetone	983.1 ± 6.9^a^	88.1
Dimethyl sulfoxide	902.3 ± 33.5^a^	80.9
Acetonitrile	1038.1 ± 7.7^a^	93.1
Surfactant (5%)		
Tween 20	1316.1 ± 39.3^a^	118.0
Tween 40	1557.9 ± 55.4^a^	139.7
Tween 60	1555.7 ± 81.4^a^	139.5
Tween 80	1582.4 ± 20.8^a^	141.9
Triton X-100	2120.9 ± 40.8^a^	190.1
SDS	1468.6 ± 44.6^a^	131.7

The enzyme was incubated with various organic solvents and surfactants at 50 °C for 1 h in 50 mM Tris–HCl pH 8.0, and the residual activity was measured according to the standard method. All data are mean values ± standard deviations of triplicate measurements

^a^The difference is significant compared with the control (*P* < 0.05)

All the tested surfactants including Tween 20, Tween 40, Tween 60, Tween 80, Triton X-100, and SDS activated the enzyme activity (Table 1). Triton X-100 was found to greatly enhance the enzyme activity to 190.1% (Table 1).

### Substrate specificity and kinetic parameters of McCut

The substrate specificity of McCut was investigated using *p*-nitrophenol (*p*NP) esters and triglycerides with acyl chain lengths ranging from C_2_ to C_16_ (Table 2). The highest specific activity was observed with C_4_ substrates, *p*-nitrophenyl butyrate (*p*NPB), and tributyrin, showing the specific activity of 1147.9 and 361.1 U/mg, respectively. It efficiently hydrolyzed *p*-nitrophenyl hexanoate (*p*NPH), which is slightly weaker than that of *p*NPB, and its activities for these substrates did not decrease as the acyl chain lengths increase from 8 to 16. In contrast, substrate specificity of McCut towards triglycerides showed a different trend. The enzyme activity declined sharply with the increase of acyl chain lengths, and almost negligible activity was detected with C_14_ and C_16_ triglycerides. McCut displayed better binding affinity towards *p*NPH (*K*_m_ = 0.27 mM) than *p*NPB (*K*_m_ = 0.66 mM), while the catalytic constant (*k*_cat_) was the same for *p*NPH and *p*NPB, which was 0.46 s^−1^.

**Table 2:** Substrate specificity of McCut

Substrate	Specific activity (U/mg)	Relative activity (%)
*p*NP esters^a^		
*p*NPA (C_2_)	553.5 ± 2.5^EF^	48.2
*p*NPB (C_4_)	1147.9 ± 8.3^A^	100
*p*NPH (C_6_)	1076.2 ± 5.0^B^	93.7
*p*NPC (C_8_)	646.9 ± 33.3^D^	56.3
*p*NPD (C_10_)	607.6 ± 28.3^DE^	52.9
*p*NPL (C_12_)	494.5 ± 5.0^F^	43.1
*p*NPM (C_14_)	962.6 ± 5.0^C^	83.8
*p*NPP (C_16_)	619.5 ± 10.8^DE^	54.0
Triglycerides^b^		
Triacetin (C_2_)	183.6 ± 4.8^B^	50.8
Tributyrin (C_4_)	361.1 ± 11.6^A^	100
Tricaproin (C_6_)	175.7 ± 9.1^B^	48.7
Tricaprylin (C_8_)	30.7 ± 2.9^C^	8.5
Tricaprin (C_10_)	10.2 ± 0.5^D^	2.4
Trilaurin (C_12_)	7.9 ± 0.4^D^	2.2

All data are mean values ± standard deviations of triplicate measurements

Mean values associated with different capital letters are siginificantly different within *p*NP esters or triglycerides

^a^Activities of McCut towards *p*NP esters were measured at 45 °C in 50 mM Tris–HCl pH 8.0

^b^Activities with triglycerides as substrates were performed at 45 °C in 2.5 mM Tris–HCl pH 8.0 containing 0.1% (w/v) Triton X-100 and 0.1% (w/v) arabic gum

### Degradation of polymers by McCut

McCut could degrade cutin with a hydrolytic rate of 4.88 µmol/h/mg protein. Moreover, it hydrolyzed aliphatic polyesters polycaprolactone (PCL) and poly(butylene succinate) (PBS) with the weight losses of 55.8 and 41.3% at 12 h, respectively (Table 3). However, McCut could not degrade aromatic polyester polyethylene terephthalate (PET).

**Table 3:** Degradation of polyesters by McCut

Polyester	Degradation of weight loss percent (%)		
	3 h	6 h	12 h
PCL	13.0 ± 0.8	48.1 ± 0.1	55.8 ± 3.9
PBS	7.2 ± 0.3	20.0 ± 0.3	41.3 ± 0.6

The reaction was carried out at 45 °C in 100 mM Tris–HCl pH 8.0. The weight loss of polyesters was measured after degradation. All data are mean values ± standard deviations of triplicate measurements

### Synthesis of butyl butyrate by McCut

**Fig. 6:**
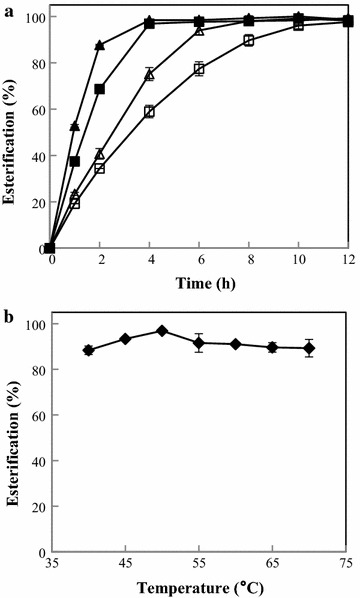
Effects of enzyme dosage (**a**) and temperature (**b**) on butyl butyrate synthesis by McCut. In Fig. 4a, reactions were performed at 50 °C, 200 rpm for 12 h, and enzyme dosages were 200 U/mL (open square), 300 U/mL (open triangle), 400 U/mL (filled square), and 600 U/mL (filled triangle). In Fig. 4b, reactions were conducted at 40–70 °C for 4 h with an enzyme dosage of 400 U/mL. The values are the average of experiments performed in triplicate

Different dosages (200–600 U/mL) of the cutinase were used in the synthesis of butyl butyrate (Fig. 6a). It took shorter time to reach equilibrium at higher enzyme dosage. The highest esterification efficiency of 96.9% was achieved at 4 h using a cutinase dosage of 400 U/mL, with the initial rate of 507.8 μmol/h/mg protein. Surprisingly, the reaction temperature had little influence on butyl butyrate synthesis by McCut from 40 to 70 °C, with a slightly higher esterification efficiency at 50 °C (Fig. 6b). McCut did not lose enzyme activity after incubation at 50 °C for 2 h, and it retained 87.7% of its initial activity after incubation for 4 h.

## Discussion

Cutinases have attracted great interest in recent years due to their great application potential in different fields, including biodiesel production, ester synthesis, textile, and detergent as well as environmental industries [9, 31]. So far, many cutinases have been cloned and expressed to meet industrial requirements for large-scale production [8, 13–16]. However, expression levels of cutinases are generally low. Here we first cloned a novel cutinase gene (*McCut*) from a thermophilic fungus *M. cinnamomea* and successfully over-expressed in *P. pastoris*. Since the yeast is adaptable for large-scale fermentation for the production of recombinant proteins, high-cell density fermentation was carried out in a 5-L fermentor. The cutinase activity reached a maximum of 12, 536 U/mL with a protein concentration of 10.8 g/L. Many cutinases from *Alternaria brassicicola* [32], *A. niger* [33], *G. cingulata* [15], and *Sirococcus conigenus* [34] have been expressed in *P. pastoris*. Among those, the production yield of *G. cingulata* cutinase is the highest, with a protein concentration of 3.8 g/L and an enzyme activity of 434 U/mL [15], which are much lower than those observed in this study. Some cutinases expressed in other hosts were also performed in bioreactor systems for scale-up production [8, 13, 14, 16]. The cutinase gene from *T. fusca* was expressed in *E. coli* with a yield of 5.1 g/L cutinase (2, 258.5 U/mL) in a 3-L fermentor, which is the highest activity level ever reported [16]. Overall, the production level reported in this study represents the highest activity for a cutinase to date, making McCut a potential cutinase for industrial-scale production.

The optimal pH of McCut (pH 8.0) is similar to those of some microbial cutinases [8, 32, 35], but is higher than those of acidic fungal cutinases, such as those from *T. terrestris* CAU709 (pH 4.0) [11, 12], *A. niger* (pH 5.0–6.5) [33], and *S. conigenus* (pH 4.1–5.2) [34]. Most alkaline cutinases display stability under near-neutral or alkaline conditions [32], whereas McCut showed excellent stability over a wider pH range from acidic to alkaline. Its stability in acidic pH values is desirable in ester hydrolysis as the pH of the reaction system tends to decrease with the release of acids. The optimal temperature of McCut (45 °C) is higher than that of most mesophilic fungal cutinases [8, 15, 32], but lower than those of cutinases from *T. terrestris* (50 and 55 °C) [11, 12] and *A. nidulans* (60 °C) [36] as well as *T. fusca* (60 °C) [35]. Interestingly, the cutinase exhibited excellent thermostability up to 75 °C. Notably, it retained 47.5% of its activity after treatment at 100 °C for 30 min. McCut is much more thermostable than most cutinases, such as those from *A. oryzae* [22], *A. nidulans* [36], *F. solani* [22], *G. cingulata* [15], *T. alba* [37], *T. fusca* [27], and *T. terrestris* [11, 12]. McCut had a half-life of 67 min at 85 °C, which is comparable to that of the cutinase from *H. insolens* [38]. The overall structure of McCut showed a low B-factor (13.44), indicating high rigidity of the structure. Furthermore, disulfide bridges are usually believed to make considerable contributions to protein stability [39, 40]. Like cutinases from *G. cingulata* [22], *F. oxysporum* [23], and *F. solani* [21], McCut has two disulfide bonds, while AoCut has three disulfide bonds [22]. However, McCut exhibited better thermostability than AoCut [22], suggesting that the disulfide bridges may not be the only reason for its thermostability. Rigidifying flexible sites through introduction of prolines is an approach to improve thermostability of proteins by decreasing the entropy of the unfolded state [40], and it has been successfully used to enhance the thermostability of proteins including cutinases [37, 41, 42]. Structure analysis revealed that most of prolines in McCut are located in the flexible loop regions (except Pro46 in α-helix), which may contribute to its excellent thermostability. The good stability of McCut over a wide pH range, coupled with the excellent thermostability, makes it suitable for industrial use.

McCut displayed a wide range of substrate specificity with the highest activity towards C_4_ esters *p*NPB and tributyrin, which is consistent with that of other cutinases, such as Acut3-6hp from *Arxula adeninivorans* [8], CUTAB 1 from *A. brassicicola* [32], FsCut-6hp from *F. solani* f. sp. *pisi* [8], and TtCutA and TtCutB from *T. terrestris* [11, 12]. In contrast, several cutinases are most active on shorter fatty acid esters, such as cutinases from *Coprinopsis cinerea* (C_3_, followed by C_2_) [14] and *S. conigenus* (C_2_, followed by C_3_) [34], while cutinases Acut1-6hp and Acut2-6hp from *A. adeninivorans* (C_6_) prefer longer chain fatty acid esters [8]. In general, substrate preference towards short acyl chain length (≤6) is a typical characteristic of cutinases, except *G. cingulata* cutinase, which preferentially hydrolyzes medium- and long-chain *p*NP esters (C_8_–C_14_) [15]. It is worth noting that the specific activity of McCut towards various *p*NP esters did not show any significant decrease with carbon chain lengths increasing from 8 to 16, which is distinguished from the behavior of most other cutinases [8, 11, 12, 14, 34]. The large and hydrophobic residues are crucial to increase the specificity of cutinases towards long-chain length substrates [9]. Hydrophobic aromatic residues Phe52, Tyr145, and Phe174 located close to the catalytic triad are important for the specificity of CLE towards the long-chain substrates [43], while superimposition of the structures of McCut and CLE showed that there is no hydrophobic aromatic residue in the catalytic groove in McCut. In addition, the space steric hindrance caused by Ser34 may lead to its preference for short-chain substrates.

McCut efficiently degraded cutin, the rate of which is higher than that of cutinases from *T. terrestris* (3 µmol/h/mg protein) [12], and *T. fusca* (4 µmol/h/mg protein) [35], but lower than LC-cutinase (6 µmol/h/mg protein) from a metagenomics library [44]. McCut could hydrolyze aliphatic polyesters PCL and PBS. The hydrolysis efficiency of PCL by McCut is comparable to that of the cutinases from *A. brassicicola* (43%) and *F. solani* (50%) [38]. In contrast, AoCut and cutinases from *A. fumigatus* and *H. insolens* completely hydrolyzed PCL in 6 h [38]. The close distance between the two gatekeeper residues (Fig. 4c) and the space steric hindrance created by Ser34 (Fig. 4b) in McCut may explain its poor degradation ability on PCL in comparison with that of AoCut [22, 38]. A model for the binding of TfCut2 from *T. fusca* towards polymeric substrate indicated that an aromatic clamp formed by Tyr60 and Trp155 is a key element responsible for the high activity towards PET [27]. Therefore, no hydrophobic residue in catalytic groove may be the reason that McCut could not hydrolyze aromatic polyester PET.

Butyl butyrate is a valuable fuel source which possesses excellent compatibility with aviation kerosene, petrol, and diesel [2, 3]. To date, butyl butyrate has been successfully synthesized by several lipases through enzymatic routes [4–7]. Cutinases may be more attractive for butyl butyrate production since they prefer short-chain length substrates containing 4–6 carbon atoms and usually achieve high yields [45, 46]. McCut displayed extremely good stability in the presence of alkanes (Table 1), which are commonly used as the reaction media in ester synthesis [47]. Furthermore, the preference of McCut for C_4_ substrates may also make it more suitable for butyl butyrate synthesis. Hence, the application potential of McCut in butyl butyrate was evaluated. McCut efficiently synthesized butyl butyrate with an esterification efficiency of 96.9%, which is comparable to those of the cutinases from *T. terrestris* [11], *Burkholderia cepacia* [45], and *F. solani pisi* [46], as well as the lipases from *Thermomyces lanuginosus* [4] and *Rhizomucor miehei* [7]. Nevertheless, the reaction time of 4 h is much shorter than those obtained by other enzymes [4, 7, 11, 45]. Moreover, McCut exhibited excellent stability during reaction. Therefore, McCut may be a good candidate for in situ esterification for butyl butyrate production in biofuel industry.

## Conclusions

A cutinase gene (*McCut*) from *M. cinnamomea* was cloned and over-expressed in *P. pastoris* for the first time. The cutinase yield is the highest value obtained to date, indicating great potential for industrial production. The cutinase exhibited excellent pH and thermal stability, as well as broad substrate specificity. In addition, it synthesized butyl butyrate with high efficiency. These excellent properties make the cutinase potentially useful in butyl butyrate production in biofuel and chemical industries. Moreover, the structure of the enzyme provides valuable information for enhancing catalytic performance on polyesters, which may find use in biodegradation.

## Methods

### Reagents

T4 DNA ligase and restriction endonucleases were from New England Biolabs (Ipswich, MA, USA). LA Taq DNA polymerase was purchased from TaKaRa (Dalian, China). *p*-Nitrophenyl acetate (*p*NPA), *p*NPB, *p*-nitrophenyl caprylate (*p*NPC), *p*-nitrophenyl decanoate (*p*NPD), *p*-nitrophenyl laurate (*p*NPL), *p*-nitrophenyl myristate (*p*NPM), *p*-nitrophenyl palmitate (*p*NPP), and *p*NP were purchased from Sigma Chemical Company (St. Lous, MO, USA). *p*NPH was from HEOWNS Company (Tianjin, China). All other chemicals used were of analytical grade unless otherwise stated.

### Strains and media

*Escherichia. coli* strain DH5α [F^−^, φ80d*lac*ZΔM15, Δ(*lacZYA*-*argF*)U169, *deoR*, *recA*1, *endA*1, *hsdR*17(r_K_^−^, m_K_^+^), *phoA*, *supE*44, λ^−^, *thi*-1, *gyrA*96, *relA*1], and *P. pastoris* GS115 (*his4*) were used as hosts for gene cloning and expression, respectively. *M. cinnamomea* S168 was deposited in the China General Microbiological Culture Collection Center under Accession No. 6022. The liquid culture medium contained the following (g/L): glutinous rice flour 15.0, tryptone 8.0, yeast extract 8.0, KH_2_PO_4_ 1.0, NaCl 0.5, MgSO_4_·7H_2_O 0.5, FeSO_4_·7H_2_O 0.01, CaCl_2_ 0.2, and MnSO_4_·7H_2_O 0.05, natural pH.

Fermentation basal salts (FBS) medium contained the following (g/L): CaSO_4_, 0.93; K_2_SO_4_, 18.2; MgSO_4_·7H_2_O, 14.9; KOH, 4.13; glycerol, 40.0; 85% H_3_PO_4_, 26.7 mL/L. PTM_1_ trace salts contained (g/L): CuSO_4_·5H_2_O, 6.0; NaI, 0.08; MnSO_4_·H_2_O, 3.0; Na_2_MoO_4_·2H_2_O, 0.2; H_3_BO_3_, 0.02; CoCl_2_, 0.5; ZnCl_2_, 20.0; FeSO_4_·7H_2_O, 65.0; biotin, 0.2; and H_2_SO_4_, 5.0 mL/L.

### Cloning and sequence analysis of a cutinase gene

For isolation of genomic DNA, *M. cinnamomea* S168 was cultured at 37 °C for 3 days with a rotation speed of 200 rpm. Fungal mycelia were collected by centrifugation at 11, 510*g* for 10 min, then washed twice with sterilized water, and ground to powder in liquid nitrogen. The genomic DNA was extracted using a Fungal DNA Midi Kit (Omega Biotek, Doraville, GA, USA). The total RNA was isolated with Trizol reagent (Invitrogen, Carlsbad, USA) and mRNAs were purified using the Oligotex mRNA Midi kit (Qiagen, Germany). First and second strands of cDNA were synthesized using PrimeScript™ RT-PCR Kit (TaKaRa, Tokyo, Japan).

The specific primers McCutF (5′-ATGAAGATCCAATTTGTTATTTCCGC-3′) and McCutR (5′-TTACGAGAGTCTATCCTCAAGCC-3′) were designed. To obtain the full-length gene and coding sequence, PCR was performed using genomic DNA and cDNA as templates, respectively. PCR conditions were as follows: a hot start at 94 °C for 5 min, 35 cycles of 94 °C for 30 s, 54 °C for 30 s, and 72 °C for 60 s, followed by a final extension step at 72 °C for 10 min. After amplification, PCR products were purified, ligated to pMD18-T vector, and sequenced.

Homology searches of nucleotides were performed using BLAST at the NCBI. Multiple alignment analysis of the amino acid sequences was carried out using the ClustalW2.0 (http://www.ebi.ac.uk/Tools/clustalw2/index.html). The signal peptide and conserved domains were analyzed at Signal P 4.1 server (http://www.cbs.dtu.dk/services/SignalP/) and ScanProsite (http://www.expasy.ch/tools/ScanProsite), respectively. *N*-Glycosylation sites were predicted using NetNGlyc 1.0 (http://www.cbs.dtu.dk/services/NetNGlyc/).

### Transformation of *P. pastoris* and expression in shake-flask

The specific primers McCutEcoRIF (5′-tgcgaGAATTCTCCCCAGTTGCAGTGGAGA-3′) and McCutNotIR (5′-tgcgaGCGGCCGCTTACGAGAGTCTATCCTCAAGCC-3′), with *Eco*RI and *Not*I sites (underlined), respectively, were used to amplify the coding region of the cutinase gene without the signal peptide sequence. PCR amplification was carried out using cDNA as the template. After digestion with *Eco*RI and *Not*I, the purified PCR product was inserted into pPIC9K, yielding the recombinant plasmid pPIC9K–McCut. The recombinant plasmid was linearized with *Sal*I and then transformed into *P. pastoris* GS115 by electroporation. The transformants were plated on MD (minimal dextrose) plates and incubated at 30 °C for 2–3 days. To screen multiple inserts, the colonies from MD plates were plated on YPD-G418 plates with G418 concentrations of 1.0, 2.0, 4.0, and 6.0 mg/mL. Then G418 resistant colonies were tested for the expression of cutinase using BMGY/BMMY according to Multi-copy *Pichia* Expression Kit (Invitrogen Inc.). After induction by methanol for 3 days, the crude enzyme was used for cutinase activity analysis.

### High-cell density fermentation

For scale-up cutinase production, the transformant showing the highest cutinase activity in shake-flask culture was cultivated in a 5-L fermentor with 1.5 L working volume at 30 °C. The fermentation process including glycerol batch phase, glycerol fed-batch phase, and methanol fed-batch phase was performed according to *Pichia* Fermentation Guidelines (Version B, 053002, Invitrogen Inc.). The fermentation medium for cutinase production in the fermentor was FBS medium containing 4.35 mL/L of PTM_1_ trace salts. Throughout the cultivation period, the dissolved oxygen level was maintained above 20% by adjusting the air flow rate and agitation speed. The initial pH was controlled at 4.0 by ammonium hydroxide. Upon depletion of initial glycerol, fed-batch fermentation was initiated with the addition of 50% (w/v) glycerol containing 12 mL/L PTM_1_ trace salts at a rate of 18.4 mL/h/L (initial medium volume). After a 6-h glycerol fed-batch phase, the methanol induction phase was started with the addition of 100% methanol containing 12 mL/L PTM_1_ trace salts at a flow rate of 10 mL/h/L (initial medium volume), and the medium pH was adjusted to 6.0. During the methanol induction phase, samples were withdrawn every 12 h, and dcw, cutinase activity, and protein content were determined. The cell-surface cutinase activity on *P. pastoris* cell wall was measured by the method of Su et al. [48]. The cells were collected by centrifugation at 11, 510*g* for 10 min, washed three times with distilled water, and resuspended in 50 mM Tris–HCl pH 8.0, and the activity of the suspended solution was then assayed according to the standard method. The dry cell weight (dcw) was quantified by drying the washed cells until a constant weight was achieved.

### Enzyme assay and protein determination

Cutinase activity was assayed by the method of Xu et al. [11] using *p*NPB as a substrate. The reaction mixture (500 μL) contained 400 μL 50 mM Tris–HCl buffer pH 8.0, 50 μL suitably diluted enzyme, and 50 μL 20 mM *p*NPB dissolved in isopropanol. After reaction for 10 min at 45 °C, the released *p*NP was quantified by measuring the absorbance at 410 nm. One unit of enzyme activity was defined as the amount of enzyme liberating 1 μmol *p*NP per min under the above conditions. Protein concentration was determined according to the method of Lowry et al. [49], using bovine serum albumin as the standard.

### Purification of McCut

The crude enzyme was collected by centrifuging the fermentation culture at 11,510*g* for 10 min. After dialysis against 20 mM phosphate buffer pH 8.0 (buffer A) for 16 h, the crude enzyme was loaded onto a Q Sepharose Fast Flow column equilibrated with buffer A at a flow rate of 0.5 mL/min. After washing with buffer A, the bound proteins were eluted with a linear NaCl gradient from 0 to 500 mM in buffer B (20 mM phosphate buffer pH 8.0) at 1 mL/min. The fractions with cutinase activity were checked for purity by SDS-PAGE.

### Crystallization, data collection, and structure determination

Crystallization experiments were set up using the sitting-drop vapor diffusion method according to Qin et al. [50]. Crystals suitable for diffraction were grown in drops containing 0.2 M KCl and 20% polyethylene glycol 3350. Diffraction data of McCut were collected at beamline BL17U at Shanghai Synchrotron Research Facility (SSRF). The data were indexed, integrated, and scaled using the program *HKL*-2000 [51].

The structure of McCut was determined by molecular replacement using the coordinates of *A. oryzae* cutinase (PDB ID: 3GBS). The structure model was built and refined with the Phenix suite [52]. The detailed statistics of data collection and refinement are shown in Table 4.

**Table 4:** X-ray data collection and refinement statistics

McCut	
Data collection statistics	
Radiation source	SSRF-BL17U
Wavelength (Å)	0.9792
Resolution (Å)	28.3–1.76 (1.823–1.76)
Space group	*P2*_*1*_ *22*_*1*_
Unit cell parameters	
*a*, *b*, *c* (Å)	34.4, 37.8, 128.0
α, β, γ (°)	90, 90, 90
Protein molecules in asymmetric unit	1
Unique reflections	16,910 (1626)
Completeness (%)	98.0
*R*_merge_^a^ (%)	6.8 (11.8)
Wilson B-factor (Å^2^)	15.65
Refinement statistics	
Resolution (Å)	1.76
*R*_work_^b^ (%)	0.1717 (0.1744)
*R*_free_^b^ (%)	0.1754 (0.1619)
RMSD	
Bond lengths (Å)	0.006
Bond angles (°)	0.85
Average B-factors (Å^2^)	13.44
Macromolecules	11.66
Ligands	–
Solvent	24.57
Ramachandran	
Most favored regions (%)	97
Additional allowed regions (%)	2.2
Disallowed regions (%)	0
Clashscore	2.22
PDB code	5 X 88

^a^ $R_{\text{merge}} = \sum _{hkl} \sum _{i} |Ii\left( {hkl} \right) - \left\langle {I\left( {hkl} \right)} \right\rangle |/ \sum _{hkl} \sum _{i} Ii\left( {hkl} \right),$ where *Ii*(*hkl*) is the *i*th observation of reflection *hkl* and *I*(*hkl*) is the weighted average intensity for all observations *i* of reflection *hkl*

^b^ $R_{{{\text{work}}/{\text{free}}}} = \sum _{hkl} \left| {\left| {\text{Fo}} \right| - {\text{k}}} \right|{\text{Fc}}\left| {\left| {/ \sum _{hkl} } \right|{\text{Fo}}} \right|;$ 95 and 5% of reflections were used for *R*_work_ and *R*_free_, respectively

### Biochemical properties of McCut

The optimal pH of McCut was determined in the pH range of 3.0–11.0. The buffers used were citrate (pH 3.0–6.0), phosphate (pH 6.0–8.0), Tris–HCl (pH 7.0–9.0), 4-(2-hydroxyethyl)-1-piperazineethanesulfonic acid (HEPES, pH 7.0–8.0), Tricine (pH 7.5–8.5), and 2-(cyclohexylamino) ethanesulfonic acid (CHES, pH 8.0–10.0). For the determination of pH stability, the enzyme was incubated at 50 °C for 30 min in the above buffers. After cooling on ice for 30 min, the residual activity was measured at 45 °C in 50 mM Tris–HCl buffer pH 8.0.

The optimal temperature for cutinase activity was evaluated by measuring its activity at different temperatures (20–55 °C) in 50 mM Tris–HCl buffer pH 8.0. To determine its thermostability, the residual activity was assayed after incubation of the cutinase at 30–100 °C for 30 min in 50 mM Tris–HCl buffer pH 8.0. The denaturation half-lives of McCut at 70, 75, 80, and 85 °C were measured by incubating the cutinase at the mentioned temperatures in 50 mM Tris–HCl pH 8.0 for 6 h. Samples were taken at intervals, and the residual activity was determined after cooling for 30 min.

The influence of organic solvents and surfactants on enzyme activity was tested by mixing cutinase with various organic solvents and surfactants at the final concentrations of 30% (v/v) and 5% (v/v), respectively. After incubation in 50 mM Tris–HCl buffer pH 8.0 at 50 °C for 1 h, the residual activity was measured by the method as described above.

### Substrate specificity and kinetic parameters of McCut

The specificity of McCut towards various *p*NP esters and triglycerides was tested at 45 °C in 50 mM Tris–HCl buffer pH 8.0 by the method of Yang et al. [12].

The kinetic parameters, *K*_m_ and *V*_max_, of McCut towards *p*NPB and *p*NPH were determined using different substrate concentrations at 45 °C in 50 mM Tris–HCl buffer pH 8.0 for 5 min. The *K*_m_ and *V*_max_ were calculated using the software GraFit.

### Degradation of polymers by McCut

The hydrolysis property of McCut on apple cutin was performed at 45 °C in 25 mM Tris–HCl pH 8.0 according to the method of Yang et al. [12].

The degradation of synthetic polyesters PCL, PBS, and PET was determined by the method of Liu et al. [22]. Approximately 35 mg polyesters were added into 2.5 mL 100 mM Tris–HCl pH 8.0 with a concentration of 0.5 mg cutinase and incubated at 45 °C, 200 rpm for 12 h. Weight losses of polyesters were calculated after drying.

### Synthesis of butyl butyrate by McCut

The synthetic reactions by McCut were carried out in 5 mL isooctane containing 0.1 M butyric acid, 0.2 M butanol, 200–600 U/mL of spray-dried cutinase powder, and 40 mg/mL molecular sieve 4 Å. The mixtures were incubated at 40–70 °C with constant shaking at 200 rpm.

The periodically withdrawn samples were analyzed using a gas chromatograph (Agilent 6890, Hewlett-Packard Co., Avondale, PA, USA), equipped with a flame ionization detector and an HP-INNOWax column (30 m × 0.32 mm × 0.25 μm). The carrier gas used was nitrogen. The temperatures of both injector and detector were maintained at 250 °C. The oven temperature was held at 50 °C for 3 min and then increased to 220 °C at a rate of 15 °C/min and held for 2 min.

The stability of McCut under the optimal reaction conditions (in isooctane containing 0.1 M butyric acid and 0.2 M butanol with an incubation temperature of 50 °C) was determined. Samples withdrawn at 1, 2, and 4 h were centrifuged at 11,510*g* for 10 min, and the precipitates (cutinase) were dissolved in 50 mM Tris–HCl pH 8.0. The residual activity was determined at 45 °C in 50 mM Tris–HCl pH 8.0.

## Abbreviations

*p*NP: *p*-nitrophenol
*p*NPA: *p*-nitrophenyl acetate
*p*NPB: *p*-nitrophenyl butyrate
*p*NPC: *p*-nitrophenyl caprylate
*p*NPD: *p*-nitrophenyl decanoate
*p*NPL: *p*-nitrophenyl laurate
*p*NPM: *p*-nitrophenyl myristate
*p*NPP: *p*-nitrophenyl palmitate
*p*NPH: *p*-nitrophenyl hexanoate
MD: minimal dextrose
BMGY: buffered minimal glycerol complex medium
BMMY: buffered minimal methanol complex medium
HEPES: 4-(2-hydroxyethyl)-1-piperazineethanesulfonic acid
CHES: 2-(cyclohexylamino) ethanesulfonic acid
